# Biocompatibility and biocidal effects of modified polylactide composites

**DOI:** 10.3389/fmicb.2022.1031783

**Published:** 2022-11-24

**Authors:** Tereza Stachurová, Zuzana Rybková, Kateřina Škrlová, Kateřina Malachová, Miroslav Havlíček, Daniela Plachá

**Affiliations:** ^1^Department of Biology and Ecology, University of Ostrava, Ostrava, Czechia; ^2^Nanotechnology Centre, VSB–Technical University of Ostrava, Ostrava, Czechia; ^3^Center of Advanced Innovation Technologies, VSB–Technical University of Ostrava, Ostrava-Poruba, Czechia; ^4^Medin, a.s., Nové Město na Moravě, Czechia; ^5^Energy Units for Utilization of Non-Traditional Energy Source (ENET) Centre, Center for Energy and Environmental Technologies (CEET), VSB–Technical University of Ostrava, Ostrava, Czechia

**Keywords:** antimicrobial effect, biofilm, cytotoxicity, polylactide composites, graphene oxide, organically modified vermiculite

## Abstract

Polylactide (PLA) materials treated with antimicrobial fillers represent a suitable alternative to the production of medical devices. Their advantage is that they can prevent the growth of microorganisms and the formation of microbial biofilms on the surface and around composites. The work is focused on the evaluation of biocompatibility and biocide effect of PLA composite films filled with vermiculite and graphene oxide modified with silver (Ag^+^ and Ag nanoparticles), hexadecylpyridinium (HDP) and hexadecyltrimethylammonium (HDTMA) cations and their degradation leachates monitored at 1–3–6-month intervals. The antimicrobial effect of the leachates was detected by microdilution methods on gram-negative (*Escherichia coli*, *Pseudomonas aeruginosa*, *Proteus mirabilis*), gram-positive (*Staphylococcus aureus*, *Streptococcus salivarius*) bacteria and yeast (*Candida albicans*). The biocidal effect of composites on biofilm formation on the surface of composites was monitored by Christensen method and autoaggregation and motility tests. The biocompatibility of the composite and the leachates was assessed by 3-(4,5-Dimethylthiazol-2-yl)-2,5-Diphenyltetrazolium Bromide (MTT) cytotoxicity assay. The evaluation of the antimicrobial effect of the leachates demonstrated that leachates of PLA composite filled with graphene oxide and Ag^+^ showed a stronger antimicrobial effect than leachates of PLA composite filled with vermiculite and Ag^+^ and Ag nanoparticles. The leachates of PLA composites containing vermiculite with HDP and HDTMA cations had a higher antimicrobial effect on G^+^ bacteria and yeast than G^–^ bacteria. Bacterial growth, biofilm formation, autoaggregation and motility of the tested bacteria were most inhibited by the composite with vermiculite and Ag^+^ and Ag nanoparticles. Even after a 6-month degradation of this composite, bacterial growth and biofilm formation continued to be strongly inhibited up to 42 and 91%, respectively. The cytotoxic effect was proved only in the leachate of the composite with vermiculite containing HDP after 6 months of its degradation. Tests evaluating the biocompatibility of materials have shown that the vermiculite is the most preferred carrier and can be used in the future to bind other compounds. The study confirmed that PLA composite filled with vermiculite and Ag^+^ and Ag nanoparticles was the most stable and effective composite with the best biocompatible and biocidal properties.

## Graphical Abstract

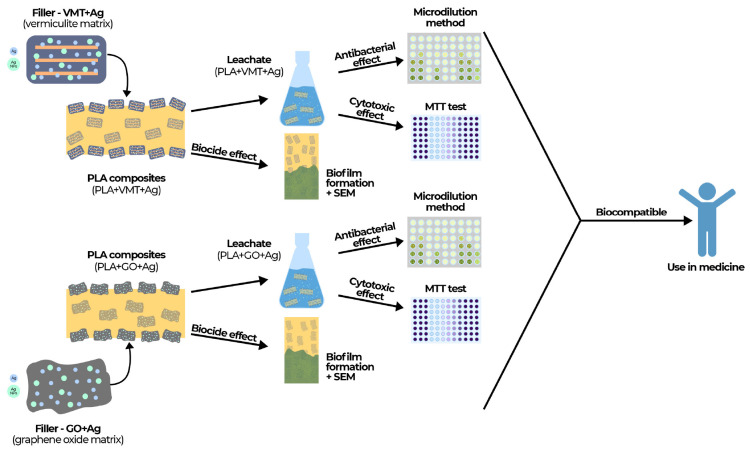

## Introduction

Medical devices used to treat patients can be contaminated with microorganisms despite strict adherence to hygiene measures. Contamination can cause problems related to prostheses and implantable devices, such as catheter sepsis, valve replacement infection, urinary catheter infections, and others (Albu et al., 2018; Gomes et al., 2018; Khatoon et al., 2018). Therefore, it is very important to search and create new materials that can prevent these problems and thus protect the patient’s health, but also reduce treatment costs.

Polylactide (PLA) (C_3_H_4_O_2_)_n_ is a hydrophobic polymer made from renewable resources. PLA belongs to the group of aliphatic polyesters. PLA is not soluble in water, immersed in water undergoes hydrolytic cleavage, which takes place on the basis of bimolecular nucleophilic substitution. This process is catalyzed in the presence of acids or bases. In an alkaline environment, oligomeric acids dissociated into RCOO- are formed. These acids are more hydrophilic than undissociated RCOOH oligomers, which are formed during degradation at neutral pH. RCOO- ions are released more easily than RCOOH, thus accelerating the degradation of PLA (Casalini et al., 2019; Škrlová et al., 2022).

PLA is a biocompatible polymer material; whose properties are comparable to conventional polymers. For this reason, their use in many different areas is widely investigated today (Valentina et al., 2018). It turns out that PLA materials have very promising potential in medicine, for example when used internally as stents. The stents are used to restore patency in tubular organs in the human body (blood vessels, esophagus, duodenum, gallbladder, pancreas, urethral or prostatic tracts) in the treatment of their various diseases (Škrlová et al., 2019, 2022).

Stents can be made of metals, permanent polymeric materials, or new degradable polymeric materials. The advantage of degradable polymer stents compared to permanent polymer or metals stents is that they do not have to be surgically removed from the patient’s body after treatment as they gradually break down into low-molecular weight substances that are further metabolized.

From the point of view of biomedical use, PLA can be modified, for example, with different antimicrobial agents, for example antibiotics, silver (Ag) or hexadecylpyridinium (HDP) and hexadecyltrimethylammonium (HDTMA) cations (Plachá et al., 2008, 2010). The advantage of our studied materials is that the filler is deposited not only on the surface of the polymer material (where the antimicrobial effect can only be short-term), but also in the polymer matrix. In this case, the degradability of PLA can be a very advantageous property bringing a long-term inhibitory effect of PLA composites and thus prevent the formation of a biofilm on the surface of the material and reduce the risk of infection (Škrlová et al., 2019, 2022; Gherasim et al., 2020).

Biofilms form a complex of bacteria capable of surviving in adverse environmental conditions, due to the composition of the extracellular polymer matrix (Hayat et al., 2019; Siddique et al., 2020). During implantation, bacteria present in the body can adhere to the implant or prosthetic medical device and develop inflammation. In such cases, bacterial biofilms are thought to play a significant role in more than 80% of bacterial infections (Raksha et al., 2020). Gram-positive and gram-negative biofilm-forming bacteria include, in particular, *Escherichia coli*, *Staphylococcus aureus*, *Staphylococcus epidermidis*, *Pseudomonas aeruginosa*, *Enterococcus faecalis*, *Klebsiella pneumoniae*, and *Proteus mirabilis*, but also others (Chen et al., 2013). Two-thirds of infections associated with implantable devices are caused by staphylococcal species, most of which are associated with *Staphylococcus aureus* (Khatoon et al., 2018). Due to the difficulty of infections caused by biofilm-forming bacteria, there is currently a need to look for new materials with biofilm-inhibiting antibacterial properties for implants and medical devices.

After exposure to suitable conditions that consist of a combination of moisture, oxygen, pH and naturally occurring microorganisms, PLA will decompose to water, carbon dioxide or lactic acid, and a minor quantity of non-toxic surplus material. These degradation products are excreted in the urine (Silva et al., 2018; Zaaba and Jaafar, 2020). For this reason too, it is very important to evaluate the biocompatible and biocidal effect of PLA materials and their degradable components. The studied PLA material must be compatible with a living tissue or a living system by not being toxic and added components in material, which are excreted, are harmless (Ramot et al., 2015). Biocompatibility assessment is a complex procedure that incorporates *in vitro* tests directed at evaluating cytotoxicity (Ramot et al., 2016).

The main aim of this research was to monitor difference in PLA composites containing antimicrobial agents due to degradation of these composites in saline at different pH (pH 7 as a simulation of the environment of most organs of the human body and pH 9 as a simulation of the gallbladder environment) and to determine whether during the degradation process there was a release of composite compounds into the leachates. The antimicrobial effect of the leachates was detected. The formation of bacterial biofilm on individual composites was monitored, as well as their influence on the autoaggregation and motile properties of bacterial strains at individual times of degradation. The cytotoxic effects of composites and their leachates on A549 cell culture were evaluated.

The contribution of the research is the chosen approach of evaluation including a complex of detection assays determining the biocidal and biocompatible properties of PLA composites for their potential use in medicine.

## Materials and methods

### Preparation of polylactide composites

PLA granules (IngeoTM 4032D), were supplied by RESINEX Czech Republic s.r.o. The PLA granules have a density of 1.24 g/cm^3^, glass transition temperature (Tg) of 59°C and melting point of 160°C, molecular weight (MW) of 182,000 g/mol (Škrlová et al., 2022). Graphene oxide (GO) was prepared by a modified Hummers method (Plachá et al., 2020a). Concentrated H_2_SO_4_, KMnO_4_, graphite flakes, and concentrated H_2_O_2_ were used for preparation of GO. All compounds were supplied by Sigma-Aldrich Co. Vermiculite (VMT) Palabora from Southern Africa (GRENA a.s.), with a cation exchange capacity value of 89 cmol^(+)^/kg was chosen as the matrix for the clay fillers. For better cation exchange, monoionic form Na-vermiculite was prepared by modifying VMT with NaCl (Sigma-Aldrich, Co.).

Both, GO and VMT were modified using solutions of AgNO_3_ (> 99%), fillers based on organically modified VMT were prepared using HDP cations (purity of > 98%, Sigma-Aldrich, Co.) or HDTMA cations (purity of > 98%, Sigma-Aldrich, Co.). The modification of GO with AgNO_3_ took place in demineralized water. The solution was shaken for 24 h, and then a precipitate was allowed to form at the bottom of the beaker, which was collected and dried to a powder at 60°C. VMT was modified in all cases (AgNO_3_, HDTMA, HDP) by intercalation in an aqueous solution at 80°C for 3 h. Subsequently, the mixture was washed several times with demineralized water and dried to a powder at 60°C. Obtained fillers were denoted as GO + Ag, VMT + Ag, VMT + HDP, and VMT + HDTMA. Details of the methods of preparation as well as methods of characterizations were described several times in our previous publications (Plachá et al., 2008, 2010, 2014a,b, 2020a,b; Khammassi et al., 2022; Škrlová et al., 2022).

The fillers GO + Ag, VMT + Ag, VMT + HDP, and VMT + HDTMA as well as pure GO and VMT were consequently used for preparation of the PLA composites films. The PLA films were prepared from PLA pellets and fillers (1 wt%) were mixed together using a polymeric extruder (Thermo Scientific™ HAAKE™ MiniLab 3 Micro Compounder) at 160°C, 100 rpm and for 5 min. After, these composites were compressed at a temperature of 200°C for 5 min to form films. The PLA composite films were denoted as PLA + GO, PLA + GO + Ag, PLA + VMT, PLA + VMT + Ag, PLA + VMT + HDP, PLA + VMT + HDTMA (Škrlová et al., 2022). The content of antimicrobial agents which are anchored on GO or VMT matrices is presented in Supplementary Table 1. PLA film without any filler was also prepared in the same way to compare the pure PLA properties with properties of the composites.

The characterization of the materials used in this study was described in detail in our previous study (Škrlová et al., 2022) and is briefly presented in Supplementary Text 1 and Supplementary Table 1.

#### Preparation of samples and their degradation leachates for testing

PLA and their composites (PLA + GO, PLA + GO + Ag, PLA + VMT, PLA + VMT + Ag, PLA + VMT + HDP, and PLA + VMT + HDTMA) were cut into 6 mm diameter discs that were further used for biocompatible and biocidal tests. Specially, 8 × 4 mm samples of composites were also prepared for monitoring biofilm formation, autoaggregation and motility testing, and scanning electron microscopy (SEM) measuring. All samples were placed in 0.9% NaCl (Penta, Czech Republic, pH 7 for biofilm formation, autoaggregation and motility tests, pH 7, and pH 9 for 3-(4,5-Dimethylthiazol-2-yl)-2,5-Diphenyltetrazolium Bromide (MTT) test, indirect and direct contact assay) and degraded for 1, 3, and 6 months at 37°C. After selected time of degradation the composites were removed from the saline solution and immersed in 96% (v/v) ethanol for 30 min and then sterilized under UV light for 60 min. Changes in the biological effects of composite materials caused by the release of substances from composites due to their degradation in the interval of 1–6 months were monitored and compared. Degradation after 6 months reduced the weight of all materials by an average of 10%. The differences between the samples were not significant. The resulting leachates from composites were further tested to determine the antimicrobial and biocidal effect.

### Microbial strains

Potentially pathogen strains of *Escherichia coli* (CCM3988, *E. coli*), *Pseudomonas aeruginosa* (CCM1960, *Ps. aeruginosa*), *Staphylococcus aureus* (CCM4223, *S. aureus*), *Proteus mirabilis* (CCM7188, *P. mirabilis*), *Streptococcus salivarius* (CCM4046, *Str. salivarius*), and *Candida albicans* (CCM8186, *C. albicans*) from the Czech Collection of Microorganisms (CCM, Brno, Czech Republic) were used as reference strains for this study. Bacterial strains were routinely cultured at 37°C on the plates with Nutrient Agar (NA, HiMedia, India). Yeast strain was cultured at 28°C on the Glucose Peptone Yeast Extract (GPY) agar plates (consisting of glucose 40 g.L^–1^, peptone 5 g.L^–1^, yeast extract 5 g.L^–1^, agar 15 g.L^–1^, HiMedia, India).

### Cell culture

Human lung cancer cell line A549 was obtained from the European Collection of Cell Cultures (ECACC, Sigma-Aldrich, USA). The A549 cell line has been widely used in *in vitro* cytotoxicity studies (Tang et al., 2015). The advantages of the extensive applications of *in vitro* studies of A549 cells are their easy availability, simple cultivation and the possibility of comparing results. A549 cells were cultured at 37°C in a humidified atmosphere (95%) and 5% CO_2_ in Dulbecco’s modified Eagle medium (DMEM, Biowest, USA) supplemented with 10% fetal bovine serum (FBS, Biowest, USA) and antibiotics (streptomycin 100 μg.mL^–1^, penicillin 100 U.mL^–1^, Sigma-Aldrich, USA). The cells were grown in 75 cm^2^ tissue culture flasks and subcultured when the cells reached 70–80% cell confluence.

### Antimicrobial activities of the leachates of polylactide composites

Antimicrobial effects of the 1-, 3-, 6-month degradative leachates of PLA and its composites PLA + GO, PLA + GO + Ag, PLA + VMT, PLA + VMT + Ag, PLA + VMT + HDP, PLA + VMT + HDTMA were assessed by means of inhibition of microbial growth. Antimicrobial activities of the fillers (GO + Ag, VMT + Ag, VMT + HDP, VMT + HDTMA) and antimicrobial agents (Ag^+^, HDP, HDTMA), and pure fillers (GO, VMT) were evaluated in a previous study (Škrlová et al., 2022).

Antimicrobial analyses were performed using the standard dilution micromethod ISO 20776-1: (International Organization for Standardization [ISO], 2019). Bacterial strains were inoculated into 10 mL of Nutrient Broth (NB w/1% Peptone, HiMedia, India) and cultured overnight at 37°C. Yeast strain was inoculated into 10 mL of GPY (HiMedia, India) and cultured overnight at 28°C. After overnight incubation, microbial suspensions were diluted with a sterile 0.15 mol⋅dm^–3^ saline solution to concentrations of 1.5 × 10^8^ cfu⋅mL^–1^. A volume of 100 μL tested sample, 80 μL NB or GPY and 20 μL of suspension of microorganism was added to each well of 96-well flat-bottom polystyrene microtiter plate (Anicrin, Italy). A total of 180 μL of NB or GPY and 20 μL of microorganism suspension were used as the control. The microtiter plates were incubated at 37°C (bacteria) or 28°C (yeast) at shaking 120 rpm (ELMI orbital shaker DOC-20l, Latvia) for 24 h. After this incubation, the inoculum was transferred with the hand 48-needle inoculator (Erba Lachema, Czech Republic) to wells with 200 μL of NB or GPY and the microtiter plates were incubated 24 h at 37°C or 28°C. After incubation, the presence of turbidity was detected spectrophotometrically (Epoch microplate spectrophotometer, BioTek, USA) at a wavelength of 600 nm. All antimicrobial tests were done in six replicates. A significant antimicrobial effect (+ +) indicates complete inhibition of the growth of microorganism. The partial effect (+) represents a partial reduction in the absorbance compared to the control. The negative effect (–) means that the leachate did not inhibit microbial growth, i.e., the absorbance value was comparable to the absorbance of the control.

### Influence of polylactide composites on microbial growth and biofilm formation

Before experiment, the standard strains were incubated into NB for 24 h at 37°C or into GPY (24 h at 28°C). A standardized inoculum was prepared from an overnight culture to obtain a final optical density OD600 of 0.1. A 96-well microtiter plate containing composites was filled with 300 μL of a pre-prepared inoculum of standard strains. As a negative control, the composite was used only in NB or GPY; a microbial strain inoculum was used as a positive control. To promote biofilm formation, the plate was incubated at 37°C (bacteria) or 28°C (yeast) without shaking for 24 h. After incubation, the composite was removed from each well and the absorbance at 600 nm was measured to determine microbial growth. Subsequently, biofilm formation was monitored directly on the composites. The composites were washed three times with 300 μL of distilled water. The composites were dried at 60°C for 15 min and stained with 300 μL of 0.1% crystal violet (CV, HiMedia, India) for 15 min. The composites were washed again three times with 300 μL of distilled water. They were dried again at 60°C for 15 min. CV was dissolved by adding 300 μL of 96% (v/v) ethanol. The composites were mixed at 500 rpm for 5 min. Absorbance was measured at 570 nm with a microplate spectrophotometer (Herten et al., 2017). Biofilm formation was compared to positive controls (pure bacterial strains in NB without affecting by the composite) and defined as: OD570 < 0.1, non-forming; OD570 = 0.1–1.0, weakly forming; OD570 = 1.1–3.0, moderately forming; and OD570 > 3.0, strongly forming (Stachurová et al., 2020).

### Autoaggregation and motility test

Prior to autoaggregation and motility test, standardized strains were incubated in NB or GPY together with 5 pieces of PLA composites for 24 h at 37°C or 28°C. Standardized strains without the addition of samples were used as a positive control. Subsequently, the composites were removed and only the microbial culture was used.

For the autoaggregation test, the microbial culture was centrifuged at 4,000 rpm for 5 min. Then the microbial culture washed twice and resuspended in Phosphate Buffer Saline (PBS, Sigma-Aldrich, USA) to obtain a final optical density of 1 at 600 nm. The absorbance of the upper phase of cell suspension was measured at times 0 h, 5 h and 24 h at 600 nm. Autoaggregation was determined as the autoaggregation percentage (*%AA*) using the formula:

$$\%AA=\left[1-\left(\frac{A_{t}}{A_{0}}\right)\times 100\right]$$

where *A*_0_ was the absorbance measured at 0 h and *A*_t_ was the absorbance measured after 5 h or 24 h of the incubation (Sterniša et al., 2019). Autoaggregation was evaluated as the percentage difference compared to positive controls (pure bacterial strains in nutrient medium without affecting by the composite) as defined by: stimulation (≥ 0.01%); weak inhibition (–29.99 to 0.00%); medium inhibition (–64.99 to –30.00%); strong inhibition (–100.00 to –65.00%).

For swarming motility, 5 μL of the microbial overnight culture was placed on medium NA with 0.5% (w/v) agar. For swimming motility, 1 μL of the overnight culture was placed in medium with 0.3% (w/v) agar. The colonies size was measured in two perpendicular directions (in mm) after 24 h at 37°C or 28°C (O’May and Tufenkji, 2011). The motility was evaluated as the percentage difference compared to positive controls (pure bacterial strains in NB without affecting by the composite) as defined by: stimulation (≥ 0.01%); weak inhibition (–29.99 to 0.00%); medium inhibition (–64.99 to –30.00%); strong inhibition (–100.00 to –65.00%).

### Scanning electron microscopy monitoring of bacterial biofilms on polylactide composites

Biofilm formation was monitored by SEM on *E. coli* strain that was incubated in the NB for 24 h at 37°C. The inoculum was prepared from an overnight culture to obtain a final optical density OD600 of 0.1. PLA composites were incubated with the inoculum. After cultivation, the samples were rinsed twice with PBS and the bacterial biofilm was fixed in 3% glutardialdehyde (Sigma-Aldrich, USA) in PBS for 20 min at 37°C. After dehydration through an ascending series of ethanol ending in 100% ethanol, the samples were dried (Herten et al., 2017). Afterward the samples were platinum-sputtered and analyzed with a scanning electron microscope JEOL JSM-7610F Plus (JEOL, Japan) with an auto emission source.

### 3-(4,5-Dimethylthiazol-2-yl)-2,5-Diphenyltetrazolium Bromide viability assays

Effects of all PLA composites on cellular viability was determined by MTT assay *in vitro* (Mosmann, 1983; Berridge et al., 2005; Stockert et al., 2018). Discs of 6 mm diameter were immersed in 96% (v/v) ethanol for 30 min and sterilized under UV light for 60 min. The biocompatibility of the discs and degradative leachates of the composites (1, 3, 6 month) was evaluated by direct and indirect contact with human lung cancer cell line A549 ISO 10993-5 (International Organization for Standardization [ISO]., 2009; Burugapalli et al., 2016). For MTT assay, A549 cells cultured in 75 cm^2^ flasks were detached using trypsin-EDTA solution (0.25% trypsin-0.53 mM ethylenediamine-N,N,N’,N’-tetraacetic acid, EDTA, Biowest, USA) and centrifugated at 200 x g for 5 min. The pellets were resuspended in fresh medium and the cells were counted with a Bürker chamber.

Discs in the indirect contact assay were incubated according to ISO 10993-12 International Organization for Standardization [ISO]. (2012). Each disc was immersed in 1.5 mL of the DMEM for 72 h and examined using MTT assay according ISO 10993-5 International Organization for Standardization [ISO]., 2009. The obtained extracts were tested in the concentration range 10–25–50–75–100%. The A549 cells were seeded at 1 × 10^4^ cells per well and cultured overnight at 37°C in the cell incubator (Sanyo, Schoeller Instruments) in the humidified 5% CO_2_ atmosphere. After overnight cell attachment, the culture medium was replaced with the extracts of appropriate concentration (100 μL/well). In the direct contact assay, the A549 cells were seeded directly on the discs at 1 × 10^4^ cells per well. The same concentration of A549 cells was applied in the degradation leachates assay. Then, the growth medium was aspirated and 100 μL of the leachates was added to each well and incubated for 24 h at 37°C. After this exposure, the medium was from the wells aspirated and cells were washed with PBS solution. Then 100 μL of the 3-(4,5-dimethylthiazolyl-2-yl)-2,5-diphenyltetrazolium bromide (MTT, Sigma-Aldrich, USA) solution (0.5 mg.mL^–1^) was added to each well and the plates were incubated in the cell incubator for 2 h. The yellow water-soluble MTT substrate is converted by the metabolically active cells to a water-insoluble purple formazan. Subsequently, the solutions in the wells were replaced with 100 μL of the dimethylsulfoxide (DMSO, VWR International, France). The plates were shaken at room temperature for 20 min. until the formazan crystals had dissolved. Finally, the purple formazan was recorded at 570 nm wavelength using a microplate reader (Epoch, BioTek, USA). The optical density directly corresponds to the number of metabolically active cells. The results were assessed as the percentage of viability compared to the control (untreated cells) considered 100% viable (Râpă et al., 2020). The sample is considered potentially cytotoxic if cell viability falls below 70% control viability (untreated cells) ISO 10993-5 (International Organization for Standardization [ISO]., 2009; Liu et al., 2018). The samples were tested in six parallels and data were expressed as the mean ± standard deviation (SD).

### Statistical analysis

The level of significance between individual samples of PLA composites was determined using one-way ANOVA with Tukey’s multiple comparison test (Lade et al., 2019). *p* ≤ 0.05 was considered significant. All statistical analyses were executed using the program R (R Core Team, 2016, version 3.4.0).

## Results and discussion

### Antimicrobial activity of leachates of polylactide composites

The antimicrobial effect of leachates from PLA composites PLA + GO + Ag, PLA + VMT + Ag, PLA + VMT + HDP, and PLA + VMT + HDTMA was detected by the microdilution method ISO 20776-1: (International Organization for Standardization [ISO], 2019). G^–^ (*E. coli*, *Ps. aeruginosa*) and G^+^ (*S. aureus*, *Str. salivarius*) bacterial strains and yeast *C. albicans* were used for detection. The evaluation was performed depending on the degradation time of the composites (1, 3, 6 months) and the pH of the degradation solution (pH 7, pH 9). The determined antimicrobial effect of the degradation leachates was compared with the inhibitory effect of degraded composites on the growth of microorganisms determined by the contact test (see section “Influence of Polylactide Composites on Microbial Growth and Biofilm Formation”). Degradation leachates of pure PLA, and PLA + GO and PLA + VMT composites, in which no antimicrobial effect was detected, were used as negative controls. Under this condition, the antimicrobial effects of leachates can only be attributed to agents (Ag^+^, AgNPs, HDP or HDTMA) bound to the composite filler and released into the leachates during degradation. The results in the antimicrobial effects of the leachates showed that the antimicrobial effect depends not only on the concentration of substances bound to the fillers, but also on the type of GO or VMT filler and degradation conditions (pH 7, pH 9), including the degradation interval (1, 3, 6 months) (Table 1). The alkaline environment was monitored because the developed materials could be used for the production of stents with application in the gallbladder (Laukkarinen et al., 2007; Lee et al., 2019; Škrlová et al., 2019; Girard et al., 2021).

**TABLE 1:** Antimicrobial effect of leachates after degradation of the composites at 1, 3, and 6 months.

		Type of leachate							
		PLA + GO		PLA + VMT		PLA + VMT		PLA + VMT	
		+ Ag		+ Ag		+ HDP		+ HDTMA	
Microorganism	Time (month)	pH 7	pH 9	pH 7	pH 9	pH 7	pH 9	pH 7	pH 9
*Escherichia coli* (G^–^)	1	+ +	–	–	–	–	–	+ +	+ +
	3	+ +	+ +	++	–	+ +	–	+ +	+ +
	6	–	–	–	–	+ +	++	++	+ +
*Pseudomonas aeruginosa* (G^–^)	1	+ +	–	–	–	–	–	–	–
	3	+ +	++	++	–	–	–	–	–
	6	–	–	–	–	–	–	–	–
*Staphylococcus aureus* (G^+^)	1	+ +	–	–	–	+ +	–	+ +	+ +
	3	+ +	+ +	–	–	+ +	+ +	++	+ +
	6	–	–	–	–	+ +	++	++	+ +
*Streptococcus salivarius* (G^+^)	1	+ +	–	–	–	+ +	–	+ +	+ +
	3	+ +	+ +	–	–	+ +	+ +	++	+ +
	6	–	–	–	–	+ +	++	++	+ +
*Candida albicans*	1	+	–	–	–	+ +	–	+ +	+ +
	3	+ +	+	–	–	+ +	+ +	++	+ +
	6	–	–	–	–	+ +	++	++	+ +

+ +, strong inhibitory effect (100%); + , partial effect; –, no effect.

**FIGURE 1:**
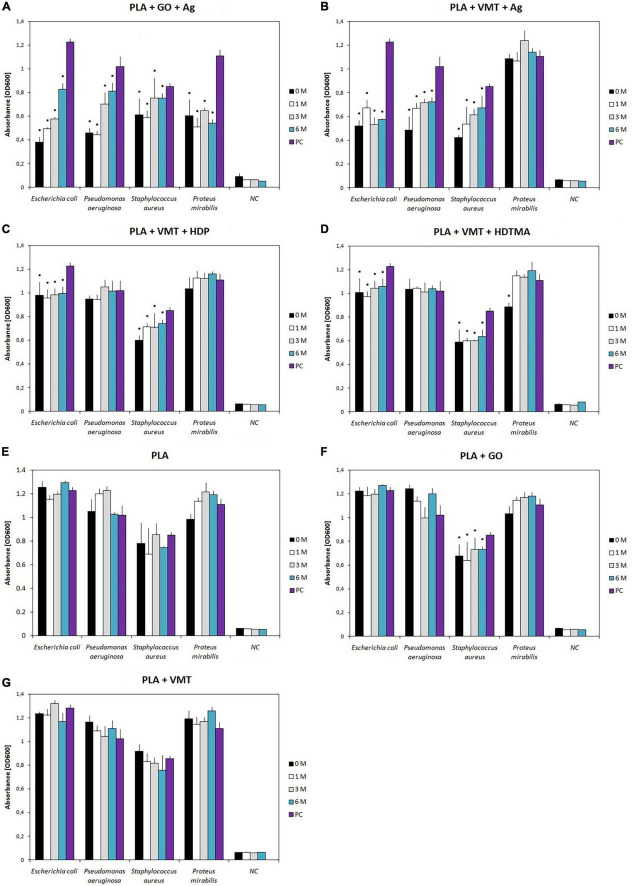
Influence of polylactide composite PLA + GO + Ag **(A)**, PLA + VMT + Ag **(B)**, PLA + VMT + HDP **(C)**, PLA + VMT + HDTMA **(D)**, PLA **(E)**, PLA + GO **(F)**, and PLA + VMT **(G)** on the microbial growth after 0, 1, 3, and 6 months of degradation of composites in saline solution. Absorbance measured after 24 h incubation at 600 nm. NC, negative control, composite sample in nutrient medium without microbial strain, PC, positive control, microbial strain in nutrient medium without composite, M, month (time of degradation). *Indicates a significant statistical difference (*p* < 0.05) between the microbial strain affected by the sample and the positive control.

According to the results of the evaluation of the antimicrobial effect of the PLA + GO + Ag leachate, it was found that at pH 7, Ag^+^ ions are released from the composite into the leachate relatively quickly. After 1 and 3 months of degradation, the inhibitory effect of the leachate was demonstrated in all bacterial strains (Table 1). These results are consistent with the evaluation of the effect of composites on the bacterial growth, when growth inhibition by the degrading composites was highest at the beginning and, conversely, gradually decreased over time (Figure 1). Only in yeast was the antifungal effect partial after 1 month with a decrease in absorbance of 57.2% and complete after 3 months. From this trend of antimicrobial effects of leachates, it could be expected that the maximum effect will be achieved in 6 months. However, the results showed that PLA + GO + Ag leaching had zero inhibitory effect after 6 months of the degradation. This could be explained by the precipitation of released Ag^+^ ions increasing with time (Besinis et al., 2014).

While the PLA + GO + Ag composite contained especially Ag^+^ ions, PLA + VMT + Ag carried Ag^+^ ions together with AgNPs (Škrlová et al., 2022). The antimicrobial effect of PLA + VMT + Ag leachate was recorded at pH 7 only in G^–^ bacteria, after only 3 months (Table 1). This finding also corresponds to the evaluation of G^–^ bacterial growth inhibition. After 1 month, there was a partial release of Ag^+^ ions, which was reflected in a reduction in the inhibitory effect of the composite on the growth of G^–^ bacteria, but in the following months, the growth inhibition did not change much (Figure 1). Both types of tests (antimicrobial activity test of leachates and growth inhibition test of composites) showed that Ag^+^ ions reached 100% antibacterial effect after 3 months. After 6 months, the effect was already null due to Ag^+^ precipitation, which manifested itself in the visible turbidity of the leachate. Thus, Ag^+^ showed impaired bioavailability similar to the study Malachová et al. (2009). Differences in the sensitivity of G^–^ and G^+^ bacteria to silver ions and to composites with the silver-containing fillers have been reported by other authors (Jaworski et al., 2018; Mohammadnejad et al., 2018; Wang et al., 2020). A comparison of the antimicrobial activity of PLA + GO + Ag and PLA + VMT + Ag leachates showed that the nature of the filler and its ability to bind a sufficient amount of silver play an important role in the release of Ag^+^ ions into the leachates. While silver is surface-bound to GO, it binds to VMT both to the surface and to the interlayers and is therefore less well extracted. In addition, the Ag content in the GO + Ag and VMT + Ag fillers as determined using ICP-AES, was 61 wt.% and 9 wt.% of Ag (Škrlová et al., 2022). This is a reason why the PLA + VMT + Ag leachate had a weaker antimicrobial effect than the PLA + GO + Ag leachate. The alkaline environment generally had a lower effect on the release of silver from the PLA + GO + Ag and PLA + VMT + Ag into leachates compared to the neutral one (Table 1).

PLA + VMT + HDP and PLA + VMT + HDTMA leachates showed a higher antimicrobial effect on G^+^ bacteria and yeast than G^–^ bacteria (Table 1). This result also correlates with the results of the evaluation of the inhibitory effect of composites on the bacterial growth (*p* < 0.05) (Figure 1) and with the sensitivity of G^+^ strains to pure HDP (MIC = 0.5 μg.mL^–1^) and HDTMA (MIC = 0.5 μg.mL^–1^). The antimicrobial effect of PLA + VMT + HDTMA leachates was recorded in all monitored degradation intervals in all strains except *Ps. aeruginosa*. This strain is resistant to PLA + VMT + HDTMA composite (*p* > 0.05) (Figure 1) because it is resistant to HDTMA alone (Nicoletti et al., 1993). The antimicrobial effect of PLA + VMT + HDP leachates was affected in the neutral environment by the length of composite degradation only in the *E. coli* strain (Table 1). Degradation in an alkaline environment was slower, as the positive effect of the leachate on G^+^ bacteria and yeast was not recorded until the 3-month degradation. Certain differences between the antimicrobial effect of PLA + VMT + HDTMA and PLA + VMT + HDP leachates (Table 1) are related to the different chemical structure of HDP and HDTMA (Lukáč et al., 2013; Mao et al., 2020), when HDP cations are more strongly attached to the vermiculite structure than HDTMA cations (Plachá et al., 2008, 2010; Škrlová et al., 2022).

### Influence of polylactide composites on microbial growth and biofilm formation

The main criterion for the selection of strains to study the effect of composites on the growth and formation of bacterial biofilms was their natural ability to massively form biofilms. From the original 5 strains were specially selected *E. coli*, *Ps. aeruginosa*, and *S. aureus* strains. Verification tests revealed that *Str. salivarius* and *C. albicans* strains belong to the weak biofilm producers. This result is in line with the results of other authors (Cvitkovitch et al., 2003; Munusamy et al., 2018). Therefore, these strains were omitted and replaced with *P. mirabilis* strain for better evaluation of biofilm-related assays.

The effect of PLA + GO + Ag and PLA + VMT + Ag composites on the microbial growth after all degradation times was studied first. The results obtained showed that *E. coli*, *Ps. aeruginosa* and *S. aureus* strains were most inhibited by the PLA + GO + Ag and PLA + VMT + Ag composites in the initial times of degradation (Figures 1A,B). Although the inhibitory effect decreased slightly with degradation length, it was maintained throughout the degradation interval. Even after 6 months, *E. coli* growth was inhibited by PLA + GO + Ag composite by approximately 36% and PLA + VMT + Ag by 42% (Figures 1A,B). Despite the lower silver content of PLA + VMT + Ag, this composite had a higher effect. This can be explained by the presence of AgNPs within the composite. The growth of *P. mirabilis* strain was also strongly inhibited by the PLA + GO + Ag composite in all time degradations, however, there was no significant inhibition by the PLA + VMT + Ag sample (Figures 1A,B). In *P. mirabilis*, it seems important what form of silver acts on the cell structure. A recent study confirmed that *P. mirabilis* may also be resistant to nanosilver (Saeb et al., 2017). Shortly, these data highlight the effectiveness of silver in the both PLA composites on the reduction of microbial growth after different times of degradation of composites. This confirms the finding that no significant difference was observed between PLA + GO + Ag and PLA + VMT + Ag (*p* > 0.05). However, a significant difference was observed between silver-containing composites and PLA, PLA + GO, and PLA + VMT composites (*p* < 0.05). These compounds showed no effect on the microbial growth (Figures 1E–G). There was no significant difference in their effect on microbial growth between PLA + VMT + HDP and PLA + VMT + HDTMA composites (*p* > 0.05). These composites moderately inhibited the microbial growth of *E. coli* and *S. aureus* strains (*p* < 0.05). Stable, approximately 20–30% growth inhibition was noted at 0-, 1-, 3-, and 6-month intervals (Figures 1C,D). A weak stimulatory growth effect in *Ps. aeruginosa* and *P. mirabilis* strains at samples PLA + VMT + HDP, PLA + VMT + HDTMA (Figures 1C,D) is not related to the presence of HDP and HDTMA in the composites. The effect agrees with the weak stimulation effect of PLA, PLA + GO and PLA + VMT samples (Figures 1E–G).

**FIGURE 2:**
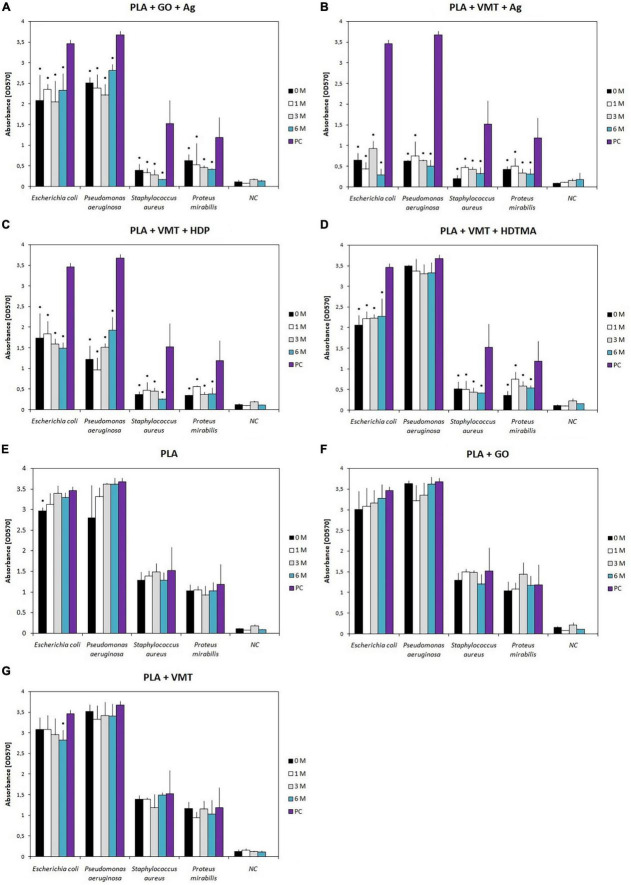
Influence of polylactide composite PLA + GO + Ag **(A)**, PLA + VMT + Ag **(B)**, PLA + VMT + HDP **(C)**, PLA + VMT + HDTMA **(D)**, PLA **(E)**, PLA + GO **(F)**, and PLA + VMT **(G)** on the biofilm formation after 0, 1, 3 and 6 months of degradation of composites in saline solution. Absorbance measured after 24 h incubation at 570 nm. NC, negative control, composite sample in nutrient medium without microbial strain, PC, positive control, microbial strain in nutrient medium, M, month (time of degradation). *Indicates a significant statistical difference (*p* < 0.05) between the microbial strain affected by the sample and the positive control.

Biofilm formation on composites after all time degradations was determined by the modified Christensen method (Sterniša et al., 2019). On the PLA + GO + Ag composite, medium to weak biofilm inhibition was observed in *E. coli*, *Ps. aeruginosa*, and *P. mirabilis* strains, which was similar at all times of degradation (*p* < 0.05) (Figure 2A). In contrast, only strain of *S. aureus*, a strong, up to 89%, inhibition of biofilm formation was observed for the PLA + GO + Ag composite (*p* < 0.05) (Figure 2A). An interesting finding was the detection of strong inhibition of biofilm formation in *E. coli*, *Ps. aeruginosa*, *S. aureus*, and *P. mirabilis* strains on the PLA + VMT + Ag composite (up to 91%) (Figure 2B). The inhibitory effect of Ag-containing PLA composites on microbial biofilm formation was also demonstrated in a study by Gherasim et al. (2020). As mentioned above, the difference between the composites can be explained by the different amount of Ag ions and AgNPs, but also in the composition of both composites. In PLA + VMT + Ag, Ag^+^ and AgNPs are found not only on the surface but also between the VMT layers, while in PLA + GO + Ag, Ag^+^ are bound mainly on the surface of GO. Comparison of the effects of the PLA + GO + Ag composite with PLA + VMT + Ag confirmed the overall stronger inhibitory effect of PLA + VMT + Ag compared to PLA + GO + Ag on biofilm formation (*p* < 0.05). This result corresponds to a smaller amount of silver released from the PLA + VMT + Ag composite compared to PLA + GO + Ag and thus also to weaker effects of PLA + VMT + Ag leachate on the tested microbial strains (Table 1). A significant difference was confirmed between the both silver-containing composites and also PLA, PLA + GO and PLA + VMT composites (*p* < 0.05) that showed no inhibition effect on the biofilm formation (Figures 2E–G). These results were also confirmed by SEM analysis of the *E. coli* biofilm on the surfaces of the silver-containing composites (not shown). Medium to strong inhibition of biofilm formation was detected in *E. coli*, *S. aureus*, and *P. mirabilis* on PLA + VMT + HDP and PLA + VMT + HDTMA composites (Figures 2C,D). In these strains, the differences in biofilm inhibition between PLA + VMT + HDP and PLA + VMT + HDTMA composites were not statistically significant (*p* > 0.05). However, in the *Ps. aeruginosa* strain, there was no inhibition of the biofilm by the PLA + VMT + HDTMA composite. The information obtained is consistent with the stimulation of bacterial growth of *Ps. aeruginosa* (Figure 1) and by the negative inhibitory effect of the leachates by the same composite (Table 1). The effect of different composite degradation times on biofilm formation was not confirmed in any of the samples (*p* > 0.05).

#### Evaluation of parameters related to biofilm formation

##### Autoaggregation test

The aggregation of bacteria is one of the essential processes that play an important part in the surface adhesion and biofilm formation (Jałowiecki et al., 2018). After the influence of the PLA + GO + Ag composite, the autoaggregation properties of *E. coli* and *Ps. aeruginosa* were stimulated, while *S. aureus* and *P. mirabilis* strains reduced autoaggregation (Supplementary Table 2). This stimulating effect over time may be related to the relatively easy extraction of Ag^+^ ions from the PLA + GO + Ag surface into the leachate, which confirmed the evaluation of the antimicrobial effect of the PLA + GO + Ag leachate (Table 1). In contrast, the PLA + VMT + Ag composite had a strong inhibitory effect on aggregation in all strains (Supplementary Table 2). This result of the effect of the PLA + VMT + Ag composite on bacterial autoaggregation is consistent with a stronger inhibition of biofilm formation by the same composite (Figure 2B). A positive correlation between bacterial biofilm and autoaggregation has been described previously (Sorroche et al., 2012). Comparison of the effects of the PLA + GO + Ag composite with PLA + VMT + Ag confirmed the overall stronger inhibition effect of PLA + VMT + Ag compared to PLA + GO + Ag on the ability to autoaggregation (*p* < 0.05). Inhibition of autoaggregation was mediated by PLA + VMT + HDP and PLA + VMT + HDTMA in all strains (Supplementary Table 2). In contrast, stimulation of autoaggregation properties was found in *Ps. aeruginosa* strain after effect of PLA + VMT + HDTMA (Supplementary Table 2). The differences in influencing the autoaggregation of the PLA + VMT + HDP and PLA + VMT + HDTMA composites were statistically significant (*p* < 0.05).

##### Motility test

The motility is a decisive factor in whether the bacteria remain adhered on the surface as a part of a biofilm or not (Jałowiecki et al., 2018). Swarming motility was inhibited in *E. coli*, *Ps. aeruginosa*, and *P. mirabilis* strains after the action of PLA + GO + Ag and PLA + VMT + Ag composites (Supplementary Table 2). This finding correlates with our results confirming the strong inhibition of biofilm by these composites (*p* < 0.05), but also with previous studies that swarming motility is directly associated with weak biofilm formation (Guttenplan and Kearns, 2013). The PLA + VMT + HDP composite had an inhibitory effect on swarming motility in *E. coli* and *Ps. aeruginosa*, while it stimulated swarming motility in *P. mirabilis* (Supplementary Table 2). However, for the PLA + VMT + HDTMA composite, the effect was the opposite (*p* < 0.05) (Supplementary Table 2). Another observed property was swimming motility. On the contrary of swarming, swimming has the opposite effect, is individual and does not have a strong effect on biofilm formation (Yang et al., 2017). The effect of composites on swimming motility was only stimulating in all samples (Supplementary Table 2). The highest stimulation was demonstrated by the composite PLA + VMT + Ag (up to 153% for *P. mirabilis*) compared to PLA + GO + Ag (up to 99% for the same strain) (*p* < 0.05) (Supplementary Table 2). In our case, *S. aureus* strain did not show motile properties (Supplementary Table 2). This can be explained by the fact that *S. aureus* is a non-motile bacterial strain and does not use swimming and swarming motility (Pollitt and Diggle, 2017; Samad et al., 2017).

### 3-(4,5-Dimethylthiazol-2-yl)-2,5-Diphenyltetrazolium Bromide viability assays

The effect of PLA, PLA + GO, PLA + GO + Ag, PLA + VMT, PLA + VMT + Ag, PLA + VMT + HDP, PLA + VMT + HDTMA and their degradation leachates (after 1, 3, 6 months) on the viability of the A549 cell line was evaluated by *in vitro* MTT assay (Mosmann, 1983; Supplementary Table 3). The cytotoxic effect on A549 cell viability was not demonstrated in any of the composites, neither by the extraction and contact MTT assay (Supplementary Table 3). The results of the cytotoxicity evaluation of the degradation leachates showed that the leachates obtained at pH 7 and pH 9 did not significantly reduce cell viability (*p* > 0.05). The only exception was the leachate of the PLA + VMT + HDP obtained after 6 months of degradation. This leachate induced a sharp decrease in viability of 92.4% in neutral and 91.8% in alkaline (*p* < 0.05). Thus, cell viability decreased significantly to 7.6 ± 0.62% (pH 7) and to 8.2 ± 0.53% (pH 9) (Supplementary Table 3). These results show that during the 6-month degradation of the PLA + VMT + HDP composite, a toxic HDP concentration was released into the leachate. From the calibration curve, determined on the basis of cytotoxicity evaluation in the interval from 0.25 to 3 μg.mL^–1^ of pure HDP (*R*^2^ = 0.9911), it can be estimated that approximately 2.5 μg.mL^–1^ HDP was extracted from the composite into the leachate. The cytotoxic potential of HDP has also been reported by other authors (Mûller et al., 2017; Araujo et al., 2020; Kanno et al., 2020).

## Conclusion

In this study, the biocompatible and biocidal effects of PLA composites based on VMT and GO fillers enriched with antimicrobial components Ag^+^, AgNPs, HDP and HDTMA and their leachates after degradation were monitored. In the PLA + GO + Ag composite, silver ions were released into the leachate more rapidly after degradation than in PLA + VMT + Ag, where AgNPs are more tightly bound in the layers of the filler. The antimicrobial effect of the PLA + GO + Ag leachate was significantly higher compared to the leachates of other composites. The corresponding finding was that the degraded PLA + GO + Ag composite showed the most inhibitory effect already after 1 month of the degradation. The study also confirmed that Ag^+^ precipitated in the leachates after 6 months of the degradation of the composites, thus reducing the inhibitory antimicrobial effects of the leachates of the silver-containing composites. PLA + VMT + HDTMA and PLA + VMT + HDP leachates showed a high inhibitory effect on G^+^ bacteria and yeast but did not inhibit *Ps. aeruginosa*. PLA + VMT + HDP leachate was also highly toxic, strongly decreased the viability of A549 cells by approximately 92% after 6 months of the composite degradation. The use of PLA + VMT + HDTMA and PLA + VMT + HDP for medicine is therefore problematic. Of all the PLA + VMT + Ag composites evaluated, it had the highest inhibitory effect on the biofilm formation. The degradation time of this composite did not affect the inhibitory effect on the biofilm formation, which is important especially in the long-term treatment of patients. From the achieved results, which are clearly summarized in Table 2, it can be assumed that the PLA + VMT + Ag composite is characterized by the best biocompatible and biocidal properties, and therefore has good preconditions for use in medicine. The study showed that in the case of composites developed for medical use, it is highly necessary to comprehensively evaluate, in addition to their physical properties such as stability and degradability, also their antimicrobial and cytotoxic effects.

**TABLE 2:** The summary of the results evaluating antimicrobial, biofilm-inhibitory and biocidal effects of studied composites PLA + GO + Ag, PLA + VMT + Ag, PLA + VMT + HDP, PLA + VMT + HDTMA and their leachates.

Composites	*E. coli*		*Ps. aeruginosa*		*S. aureus*		*P. mirabilis*		A549
	Antimicrobial effect	Biofilm-inhibitory effect	Antimicrobial effect	Biofilm-inhibitory effect	Antimicrobial effect	Biofilm-inhibitory effect	Antimicrobial effect	Biofilm-inhibitory effect	Biocidal effect
PLA + GO + Ag	+ +	+	+ +	+	+	+ +	+	++	–
PLA + VMT + Ag	+ +	++	++	+ +	++	++	–	+ +	–
PLA+VMT+HDP	+	++	–	++	+	++	–	++	–
PLA + VMT + HDTMA	+	+	–	–	+	++	–	+	–

**Leachates**	***E. coli***	***Ps. aeruginosa***	***S. aureus***	***Str. salivarius***	***C. albicans***	**A549**			
					
	**Antimicrobial effect**					**iocidal effect**			

PLA + GO + Ag	+ +	++	++	+ +	++	–			
PLA + VMT + Ag	+	+	–	–	–	–			
PLA + VMT + HDP	+ +	–	+ +	+ +	++	++			
PLA + VMT + HDTMA	+ +	–	+ +	+ +	++	–			

+ +, strong effect; + , partial effect; –, no effect.

## Data availability statement

The original contributions presented in this study are included in the article/Supplementary material, further inquiries can be directed to the corresponding author/s.

## Author contributions

KM designed the experiments. KŠ prepared the samples. TS and ZR performed the experiments. TS analyzed the experimental data. TS, KM, and ZR wrote the manuscript. DP and MH revised the manuscript. All authors contributed to the article, reviewed the manuscript, and approved the submitted version.
