# Supplementary material for: Biocompatibility and biocidal effects of modified polylactide composites
Source: Front Microbiol. 2022 Nov 24;13:1031783. doi: 10.3389/fmicb.2022.1031783 (PMC9731850; doi:10.3389/fmicb.2022.1031783)
Supplement: Supplementary file 1 [file Data_Sheet_1.docx]

Supplementary Material

**Supplementary Text S1.** Characterization of PLA polymer materials.

The materials were characterized for their physico-chemical and thermal properties as is described in (Škrlová et al., 2022). The methods used were: molecular weight (MW) by gel permeation chromatography (GPC) (Agilent GPC PL-GPC 220), inductively coupled plasma atomic emission spectrometry (ICP-AES) (SpectroCiros Vision), analysis of phase carbon (LECO RC612), Fourier transform infrared spectroscopy (FTIR) (Nicolet iS50 FTIR spectrometer (ThermoScientific, USA)) with attenuated total reflection (ATR), Raman spectroscopy (Dispersion Raman Microscope (Thermoscientific, USA)), X-ray diffraction (XRD) (RIGAKU Miniflex600 X-ray diffractometer), thermogravimetric analysis (TGA) (SETSYS Evolution (Setaram Instrumentation)), differential scanning calorimetry (DSC) (Setaram Instrumentation DSC131 EVO), analysis by optical microscopy (VHX-2000 Light Digital Microscope (Keyence Corporation, Japan)) and scanning electron microscope (SEM) (JEOL JSM-7610F Plus (JEOL, Japan)) with EDX (EDX ULTIM MAX 65 mm^2^, Oxford Instruments).

XRD analysis showed the diffraction pattern of GO+Ag with diffraction peaks at 29 39.1°, 43.4° and 64.8°, and VMT+Ag with diffraction peaks at 38.2°, 44.6° and 60.1°, which are characteristic of the presence of Ag nanoparticles. For VMT+HDP and VMT+HDTMA, the reflections were at 7.3°, 8.8°, and around 26.2°, corresponding to modified clay minerals. At the same time, XRD analysis showed the existence of a low degree of crystallinity, and PLA and PLA composites showed a more amorphous character suitable for degradation. The thermal properties of were characterized by TGA and DSC. The thermal properties of PLA composites do not differ significantly from those of pure PLA (Škrlová et al., 2022).

**Supplementary Table S1.** The composition of polylactide composites with antimicrobial and biocidal properties. The polymers were prepared with respect to the expected differences in the binding of individual biologically active components to GO and VMT fillers and differences in their degradability.

| **Composite** | **Filler** | **Amount of filler** | **Antimicrobial agent** | **Content pf antimicrobial agent** |
| --- | --- | --- | --- | --- |
|  |  | **[% wt]** |  | **[mg/100 g PLA composite]** |
| PLA | -- | -- | -- | 0 |
| PLA+GO | GO | 1 | -- | 0 |
| PLA+VMT | VMT | 1 | -- | 0 |
| PLA+GO+Ag | GO+Ag | 1 | Ag^+^ | 610 |
| PLA+VMT+Ag | VMT+Ag | 1 | Ag NPs, Ag^+^ | 90 |
| PLA+VMT+HDTMA | VMT+HDTMA | 1 | HDTMA^+^ | 299 |
| PLA+VMT+HDP | VMT+HDP | 1 | HDP^+^ | 242 |

**Supplementary Table S2.** Percentage difference of autoaggregation, swarming motility and swimming motility (%) compared to positive controls. Pure bacterial strains in nutrient medium without affecting by the composite were used as positive controls. Microbial strains affected by the polylactide composites after 0, 1, 3 and 6 months of degradation of composites in saline solution. Motility measured after 24 hours incubation at 37 °C. Autoaggregation measured at 600 nm after 24 hours incubation at 37 °C. EC - *Escherichia coli*, PA - *Pseudomonas aeruginosa*, SA - *Staphylococcus aureus*, PM – *Proteus mirabilis*, M – month (time of degradation).

| **Microbial strain** | **Composite sample** | **Autoaggregation [%]** | | | | **Swarming [%]** | | | | **Swimming [%]** | | | |
| --- | --- | --- | --- | --- | --- | --- | --- | --- | --- | --- | --- | --- | --- |
|  |  | 0 M | 1 M | 3 M | 6 M | 0 M | 1 M | 3 M | 6 M | 0 M | 1 M | 3 M | 6 M |
| *EC* | PLA+GO+Ag | 19.52 | 21.00 | 26.41 | 28.42 | 0.00 | -4.62 | -4.55 | -5.75 | 21.75 | 35.72 | 49.56 | 53.19 |
|  | PLA+VMT+Ag | -13.42 | -36.19 | -59.00 | -62.86 | -9.35 | -9.66 | -9.55 | -9.46 | 24.01 | 38.96 | 49.96 | 52.34 |
|  | PLA+VMT+HDP | -11.68 | -13.01 | -19.17 | -20.05 | -17.85 | -18.97 | -20.64 | -23.95 | 18.64 | 38.96 | 49.66 | 57.33 |
|  | PLA+VMT+HDTMA | -13.44 | -15.20 | -16.51 | -25.77 | 22.64 | 20.70 | 14.60 | 10.71 | 25.14 | 29.43 | 41.35 | 48.92 |
|  | PLA | 40.19 | 43.51 | 44.05 | 44.98 | 59.43 | 58.97 | 59.09 | 60.01 | 73.33 | 75.53 | 72.23 | 75.34 |
|  | PLA+GO | 33.94 | 34.82 | 43.68 | 54.22 | 40.75 | 45.86 | 49.09 | 56.62 | 64.29 | 66.08 | 69.56 | 72.07 |
|  | PLA+VMT | 32.14 | 28.06 | 36.03 | 41.09 | 38.87 | 37.24 | 40.45 | 42.07 | 50.79 | 57.71 | 59.56 | 67.35 |
| *PA* | PLA+GO+Ag | 12.19 | 19.81 | 27.85 | 35.21 | -28.36 | -24.56 | -21.72 | -19.96 | 62.39 | 67.62 | 68.57 | 70.02 |
|  | PLA+VMT+Ag | -56.25 | -48.36 | -75.25 | -86.34 | -26.87 | -26.67 | -18.97 | -14.07 | 27.35 | 16.43 | 14.76 | 12.52 |
|  | PLA+VMT+HDP | -4.40 | -6.67 | -7.98 | -9.74 | -13.43 | -15.79 | -18.62 | -21.33 | 50.43 | 29.29 | 22.38 | 18.90 |
|  | PLA+VMT+HDTMA | 12.46 | 18.46 | 17.12 | 18.43 | 8.96 | 14.04 | 25.86 | 33.52 | 37.61 | 22.86 | 13.33 | 10.21 |
|  | PLA | 57.66 | 52.66 | 59.53 | 64.62 | 76.42 | 77.02 | 71.72 | 68.93 | 89.40 | 82.86 | 84.76 | 83.35 |
|  | PLA+GO | 44.67 | 50.43 | 52.83 | 56.23 | 62.39 | 61.75 | 68.62 | 75.13 | 88.80 | 80.71 | 86.67 | 84.01 |
|  | PLA+VMT | 47.50 | 47.02 | 49.45 | 53.24 | 54.33 | 55.79 | 58.62 | 65.09 | 65.13 | 65.00 | 65.24 | 65.23 |
| *SA* | PLA+GO+Ag | -36.99 | -34.70 | -31.31 | -29.40 | 0.00 | 0.00 | 0.00 | 0.00 | 0.00 | 0.00 | 0.00 | 0.00 |
|  | PLA+VMT+Ag | -32.05 | -25.71 | -27.40 | -26.42 | 0.00 | 0.00 | 0.00 | 0.00 | 0.00 | 0.00 | 0.00 | 0.00 |
|  | PLA+VMT+HDP | -31.81 | -30.32 | -31.02 | -32.76 | 0.00 | 0.00 | 0.00 | 0.00 | 0.00 | 0.00 | 0.00 | 0.00 |
|  | PLA+VMT+HDTMA | 5.09 | -1.35 | -9.22 | -15.93 | 0.00 | 0.00 | 0.00 | 0.00 | 0.00 | 0.00 | 0.00 | 0.00 |
|  | PLA | 31.71 | 28.20 | 31.55 | 42.71 | 0.00 | 0.00 | 0.00 | 0.00 | 0.00 | 0.00 | 0.00 | 0.00 |
|  | PLA+GO | 30.67 | 28.93 | 32.24 | 35.88 | 0.00 | 0.00 | 0.00 | 0.00 | 0.00 | 0.00 | 0.00 | 0.00 |
|  | PLA+VMT | 24.24 | 28.45 | 29.20 | 35.09 | 0.00 | 0.00 | 0.00 | 0.00 | 0.00 | 0.00 | 0.00 | 0.00 |
| *PM* | PLA+GO+Ag | -14.47 | -36.41 | -42.66 | -52.87 | -12.12 | -14.58 | -29.03 | -34.07 | 97.78 | 97.43 | 99.24 | 98.67 |
|  | PLA+VMT+Ag | -22.24 | -40.20 | -52.46 | -60.23 | -25.76 | -20.08 | -30.65 | -33.22 | 87.78 | 103.47 | 144.30 | 153.62 |
|  | PLA+VMT+HDP | -22.04 | -21.76 | -20.72 | -18.22 | 13.64 | 31.25 | 45.16 | 56.96 | 36.67 | 31.68 | 40.89 | 46.73 |
|  | PLA+VMT+HDTMA | -12.04 | -6.76 | -10.72 | -8.66 | -21.21 | -26.25 | -20.97 | -18.24 | 42.22 | 37.62 | 43.42 | 39.04 |
|  | PLA | 6.03 | 4.57 | 11.11 | 12.27 | -27.27 | -28.45 | -29.03 | -38.22 | 46.67 | 51.68 | 65.82 | 69.93 |
|  | PLA+GO | 4.02 | 2.58 | 1.08 | -0.71 | -13.64 | -16.25 | -25.81 | -29.06 | 36.67 | 47.82 | 45.95 | 48.24 |
|  | PLA+VMT | 7.88 | 13.17 | 15.71 | 17.85 | -24.24 | -28.33 | -27.42 | -29.13 | 38.89 | 44.75 | 48.48 | 51.92 |

**Supplementary Table S3.** Cell viability of A549 cells exposed polylactide composites and degradative leachates (1, 3, 6 months).

|  | **Cell viability (%)** | | | | | | | |
| --- | --- | --- | --- | --- | --- | --- | --- | --- |
| **Samples** | **Extract test** | **Contact test** | **Degradative leachates** | | | | | |
|  |  |  | 1 month | | 3 months | | 6 months | |
|  |  |  | pH 7 | pH 9 | pH 7 | pH 9 | pH 7 | pH 9 |
| PLA | 99.7 ± 3.08 | 93.0 ± 2.20 | 98.8 ± 3.37 | 98.5 ± 4.57 | 99.2 ± 2.56 | 99.6 ± 2.79 | 99.2 ± 3.91 | 98.9 ± 3.94 |
| PLA+GO | 99.3 ± 3.64 | 93.2 ± 3.85 | 97.0 ± 4.35 | 99.6 ± 3.20 | 99.3 ± 2.87 | 98.5 ± 3.18 | 99.4 ± 2.80 | 98.4 ± 3.38 |
| PLA+GO+Ag | 99.3 ± 3.28 | 93.6 ± 3.42 | 98.7 ± 4.53 | 97.8 ± 2.81 | 100.0 ± 3.85 | 99.5 ± 3.52 | 98.1 ± 3.08 | 98.6 ± 2.76 |
| PLA+VMT | 99.1 ± 2.56 | 93.1 ± 3.14 | 99.3± 3.33 | 98.5 ± 3.28 | 99.2 ± 3.52 | 99.3 ± 3.20 | 99.3 ± 2.95 | 99.2 ± 3.36 |
| PLA+VMT+Ag | 99.4 ± 3.60 | 94.1 ± 3.67 | 97.8 ± 3.32 | 98.9 ± 3.77 | 98.5 ± 2.30 | 99.1 ± 3.28 | 99.8 ± 2.55 | 98.7 ± 2.87 |
| PLA+VMT+HDP | 99.8 ± 3.20 | 94.3 ± 3.83 | 97.1 ± 3.28 | 98.1 ± 2.59 | 99.3 ± 2.56 | 98.5 ± 3.36 | **7.6 ± 0.62** | **8.2 ± 0.53** |
| PLA+VMT+HDTMA | 99.5 ± 3.97 | 93.1 ± 2.85 | 98.4 ± 4.33 | 98.3 ± 3.56 | 99.0 ± 3.13 | 99.1 ± 2.53 | 98.0 ± 3.19 | 98.2 ± 3.25 |
